# Supplementary material for: Time-series transcriptome analysis identified differentially expressed genes in broiler chicken infected with mixed Eimeria species
Source: Front Genet. 2022 Aug 8;13:886781. doi: 10.3389/fgene.2022.886781 (PMC9393255; doi:10.3389/fgene.2022.886781)
Supplement: Supplementary file 2 [file DataSheet1.ZIP › 7dpi_KEGG.Gsea.1625115961950/KEGG_FOLATE_BIOSYNTHESIS.html]

Details for gene set KEGG\_FOLATE\_BIOSYNTHESIS[GSEA]

|  || Dataset | TMM\_7dpi\_gct\_format\_7dpi\_gct\_format.Class\_4dpi.cls #PC\_versus\_NC.Class\_4dpi.cls #PC\_versus\_NC\_repos |
| Phenotype | Class\_4dpi.cls#PC\_versus\_NC\_repos |
| Upregulated in class | 0 |
| GeneSet | KEGG\_FOLATE\_BIOSYNTHESIS |
| Enrichment Score (ES) | -0.73447895 |
| Normalized Enrichment Score (NES) | -1.6665843 |
| Nominal p-value | 0.0049972236 |
| FDR q-value | 0.062058996 |
| FWER p-Value | 0.3982 |
Table: GSEA Results Summary

  

Fig 1: Enrichment plot: KEGG\_FOLATE\_BIOSYNTHESIS      
 Profile of the Running ES Score & Positions of GeneSet Members on the Rank Ordered List

  

| SYMBOL | TITLE | RANK IN GENE LIST | RANK METRIC SCORE | RUNNING ES | CORE ENRICHMENT || 1 | PTS | na | 2254 | 0.229 | -0.1501 | No |
| 2 | SPR | na | 3172 | 0.105 | -0.2093 | No |
| 3 | FPGS | na | 4786 | -0.079 | -0.3308 | No |
| 4 | DHFR | na | 8362 | -0.466 | -0.5519 | No |
| 5 | ALPL | na | 10554 | -0.821 | -0.5994 | Yes |
| 6 | ALPP | na | 10747 | -0.865 | -0.4730 | Yes |
| 7 | QDPR | na | 11259 | -1.021 | -0.3476 | Yes |
| 8 | GGH | na | 11584 | -1.161 | -0.1836 | Yes |
| 9 | GCH1 | na | 11802 | -1.331 | 0.0173 | Yes |
Table: GSEA details [plain text format]

  

Fig 2: KEGG\_FOLATE\_BIOSYNTHESIS      
 Blue-Pink O' Gram in the Space of the Analyzed GeneSet

  

Fig 3: KEGG\_FOLATE\_BIOSYNTHESIS: Random ES distribution      
 Gene set null distribution of ES for **KEGG\_FOLATE\_BIOSYNTHESIS**
